# Brain Atrophy Correlates with Severe Enlarged Perivascular Spaces in Basal Ganglia among Lacunar Stroke Patients

**DOI:** 10.1371/journal.pone.0149593

**Published:** 2016-02-22

**Authors:** Xiaoyu Zhang, Lingling Ding, Lei Yang, Wei Qin, Junliang Yuan, Shujuan Li, Wenli Hu

**Affiliations:** Department of Neurology, Beijing Chaoyang Hospital, Capital Medical University, Beijing, China; INSERM U894, FRANCE

## Abstract

**Background:**

Enlarged perivascular spaces (EPVS) correlate with cognitive impairment and incident dementia. However, etiologies for severe basal ganglia EPVS (BG-EPVS) are still unclear. Our aim was to investigate the independent risk factors for severe BG-EPVS in patients with acute lacunar stroke.

**Methods:**

We prospectively identified patients with lacunar stroke (diameter on DWI ≤ 20mm) from Jan 2011 to May 2015. Patients with severe BG-EPVS were identified on T2 weighted MRI. Age (± 1 year) and sex matched controls were also recruited in the same population (two controls for one case). Vascular risk factors, clinical data, EPVS in centrum semiovale (rated 0 to 4), white matter hyperintensities (WMH) (by Fazekas scale), brain atrophy (rated 0 to 6) were compared between two groups. Logistic regression was performed to determine independent risk factors for severe BG-EPVS.

**Results:**

During study period, 89 patients with severe BG-EPVS and 178 matched controls were included. Vascular risk factors did not differ between two groups. Patients with severe BG-EPVS had lower level of HbA1c and diastolic BP at admission, but presented with larger infarct size, more severe WMH (including total WMH, periventricular WMH and deep WMH) and brain atrophy. In logistic regression, brain atrophy (OR = 1.40; 95%CI 1.13, 1.73) and deep WMH (OR = 1.88; 95%CI 1.24, 2.83) were independent risk factors for severe BG-EPVS.

**Conclusions:**

Brain atrophy and deep WMH are independent risk factors for severe BG-EPVS, supporting the hypothesis that brain atrophy may be associated with the development of EPVS in basal ganglia.

## Introduction

Enlarged perivascular spaces (EPVS), or Virchow Robin spaces, are common findings in elderly population with identical signal intensities to cerebral spinal fluid (CSF) in all MRI sequences. [1] EPVS are interstitial fluid filled cavities surrounding small penetrating arterioles and venules, serve as an important drainage conduit for interstitial fluid and solute in brain. [2] It has been identified that EPVS on MRI are a marker of small vessel disease (SVD) and associated with impaired cognitive function, incident dementia, depression and sleep. [3–6]

EPVS often appear in centrum semiovale and basal ganglia. The distribution patterns of EPVS in basal ganglia are in wide range, which can appear as a single enlarged space or as hundreds of bilateral. The latter pattern has been considered as severe basal ganglia EPVS (BG-EPVS). [7] The frequency of severe BG-EPVS in elderly population is low and their etiologies may be different from single EPVS in basal ganglia. In addition, severe BG-EPVS have been proposed to correlate with parkinsonism or extrapyramidal syndrome. [8] Therefore, it is of clinical importance to understand the mechanisms of severe BG-EPVS. In earlier studies, age, hypertension and white matter hyperintensities (WMH) had been identified as risk factors for EPVS in basal ganglia, [9] suggesting that EPVS in basal ganglia may be associated with hypertensive arteriopathy. [10] However, these studies only contained small number of patients with severe BG-EPVS. Etiologies for severe BG-EPVS are still poorly understood. Whether other risk factors, such as brain atrophy, contribute to severe BG-EPVS is still unknown.

Therefore, the aim of our study was to investigate independent risk factors for severe BG-EPVS in a case-control study. We paid special attention to the relationship between severe BG-EPVS and brain atrophy. We also investigated clinical and imaging characteristics of lacunar stroke patients with severe BG-EPVS.

## Materials and Methods

### Subject Population

We prospectively identified all acute lacunar stroke patients admitted to Beijing chaoyang hospital affiliated to Capital Medical University from Jan 2011 to May 2015. Lacunar stroke was confirmed by MRI and defined as an acute round or ovoid lesion of increased signal on axial diffusion-weighted imaging (DWI) ≤ 20 mm in the distribution of a small penetrating artery. As perivascular space is a subtype of SVD, only patients with small vessel disease stroke type by the Trial of Org 10172 in Acute Stroke Trial (TOAST) classification system were included in our study. [11, 12] Patients with cardioembolic risk factors (atrial fibrillation, valvular heart disease, postcardiac valve replacement, etc) and ≥50% stenosis of responsible large artery were excluded (intracranial large arteries were assessed by MR angiography and extracranial arteries were assessed by ultrasonography). Patients with history of severe or hemorrhagic stroke were also excluded because of difficult assessment on brain atrophy. We did not include patients with other neurological diseases because many of them did not have MRI or MR angiography examination. Treatments were similar among patients according to guidelines for the early management of adults with ischemic stroke. Institutional review board of Beijing chaoyang hospital affiliated to Capital Medical University approved the study and participants provided their written informed consent to participate in this study.

**Fig 1:**
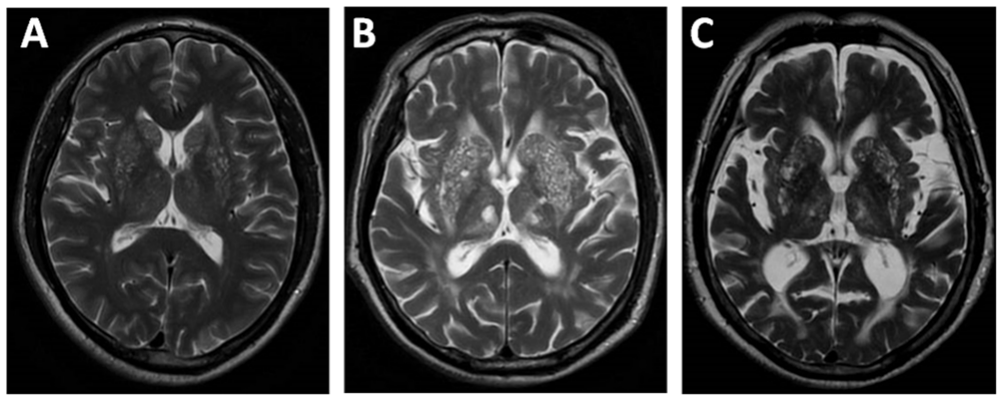
Severe basal ganglia EPVS on T2 weighted imaging. Severe basal ganglia EPVS with mild brain atrophy (A); with moderate brain atrophy (B); with severe brain atrophy (C).

Based on MRI, patients with EPVS > 40 on one side of basal ganglia were defined as severe BG-EPVS. The cut-off of > 40 EPVS was used as the highest degree of EPVS in previous studies and showed an excellent intrarater Cohen k score. [6, 13] Moreover, patients with > 40 EPVS in basal ganglia had similar neuroimages to those in Duker and Espay’s case report. [8] (Fig 1) A control group was obtained in lacunar stroke patients without severe BG-EPVS. A subset of controls was matched to cases by age (± 1 year) and sex, with two controls for each case. This was due to the fact that age and sex have been found to be associated with EPVS in basal ganglia, which could make it difficult to detect other independent contributions of EPVS. [9]

### Demographic and Clinical Assessment

We collected the following data: age, sex, and vascular risk factors including history of hypertension (defined as treatment with antihypertensive medications or BP ≥ 140/90 mmHg measured on several separate occasions), diabetes mellitus (defined as treatment with antidiabetic medications or fasting plasma glucose ≥ 7.0 mmol/L), coronary artery disease (defined as a history of myocardial infarction or angina pectoris), hyperlipidemia (based on history), stroke or transient ischemic attack (TIA) (based on history), smoking and alcohol use.

Blood pressure (BP) was recorded at admission. Results of laboratory tests including level of hemoglobin (HGB), platelets (PLT), triglyceride (TG), total cholesterol (CHOL), low density lipoprotein (LDL), high density lipoprotein (HDL), plasma fibrinogen (Fib), hemoglobin A1c (HbA1c), homocysteine (Hcy), blood urea nitrogen (BUN), serum creatinine (Cr), proteinuria (defined as positive or negative) and uric acid (UA) were recorded at admission. Stroke severity at admission was determined by NIH Stroke Scale (NIHSS).

### MRI Examinations

MR imagings were acquired on the same 3.0 T Siemens scanner (Erlangen, Germany). The parameters of MR examination were as follows: axial T1 weighted imaging (repetition time, 2000 ms; echo time, 9.2 ms; flip angle, 130°; slice thickness, 5 mm), axial T2 weighted (repetition time, 4500 ms; echo time, 93 ms; flip angle, 120°; slice thickness, 5 mm), axial diffusion weighted imaging (repetition time, 3300 ms; echo time, 91 ms; flip angle, 90°; slice thickness, 5 mm), and coronal fluid attenuated inversion recovery sequences (repetition time, 8000 ms; echo time, 86 ms; flip angle, 130°; slice thickness, 5 mm), magnetic resonance angiography (MRA) (repetition time, 23 ms; echo time, 2.8 ms; flip angle, 20°; slice thickness, 1.4 mm). All above sequences except MR angiography had 5 mm slice thickness and 1.5 mm interslice gap.

### Assessment of EPVS

Assessment of EPVS was performed by an experienced neurologist blinded to clinical information. EPVS were defined as CSF like signal intensity (hypointense on T1 and hyperintense on T2) lesions located in areas supplied by perforating arteries. They appeared round and ovoid when parallel and linear when perpendicular to the imaging plane, ≤ 3mm in their maximum diameter. We distinguished lacune from EPVS by their larger size (>3mm), spheroid shape and surrounding hyperintensity on FLAIR. EPVS in the basal ganglia and centrum semiovale were separately assessed. Patients with > 40 EPVS in basal ganglia were defined as severe BG-EPVS and the others were considered as controls. EPVS in centrum semiovale were scored by a scale which was used in a previous study: 0 = no EPVS, 1 ≤ 10 EPVS, 2 = 11 to 20 EPVS, 3 = 21 to 40 EPVS, and 4 > 40 EPVS. [6] The numbers referred to the highest number of EPVS on one side of the brain.

### Assessment of WMH and Brain Atrophy

Assessments of WMH and brain atrophy were performed by a single rater who was blinded to the hypothesis being tested. WMH was scored by Fazekas scale. [14] Periventricular WMH and deep WMH were evaluated separately and totaled together as Fazekas scores. The degree of WMH was rated by Fazekas scores (mild: 0 to 2; moderate: 3 to 4; severe: 5 to 6). Brain atrophy was assessed as deep (enlargement of the ventricles) and superficial (enlargement of the gyri) and rated by a validated scale of 0 to 3 against a reference MR brain template of normal subjects, like in a previous study. [6, 15] The degree of brain atrophy were assessed by total score (0 to 2 = mild, 3 to 4 = moderate, 5 to 6 = severe).

### Statistical Analysis

All statistics were presented as mean and SD for continuous variables with normal distribution, interquartile range for continuous variables with non-normal distribution, frequency and percentages for categorical variables. Univariate analysis was compared between patients with and without severe BG-EPVS. Continuous variables with normal distribution were compared with Student t test with significance set at p < 0.05, while Wilcoxon rank sum test for continuous variables with non-normal distribution. Categorical variables were compared by means of x^2^ test. We performed logistic regression to determine risk factors for severe BG-EPVS, with vascular risk factors, blood pressure at admission (significant by univariate analysis), laboratory tests (significant by univariate analysis), EPVS in centrum semiovale, periventricular WMH, deep WMH and brain atrophy (as continuous variable) in model. This was due to the fact that EPVS in centrum semiovale and WMH had been reported to be associated with EPVS in basal ganglia. [16] Analysis was performed with Statistical Package for Social Sciences (SPSS version 16).

## Results

During the study period, 910 lacunar stroke patients were identified. 97 patients were severe BG-EPVS. However, 2 patients with atrial fibrillation, 5 patients with large artery stenosis and 1 patient with history of severe stroke were excluded. Finally, 89 patients with severe BG-EPVS and 178 matched controls were included. The mean age of was 72.93 ± 8.47 years and 147 (55.1%) were men. 191 (71.5%) had history of hypertension, 84 (31.5%) had diabetes, 46 (17.2%) had hyperlipidemia, 50 (18.7%) had coronary heart disease, 83 (31.1%) had stroke or TIA, 107 (40.1%) were smokers and 65 (24.3%) had alcohol use. The mean infarct size was 11.48 ± 3.75mm and NIHSS scores were 3.06 ± 2.63 at admission. There are 135 (50.6%) lacunar strokes in subcortical white matter, 39 (14.6%) in basal ganglia, 35 (13.1%) in thalamus and 58 (21.7%) infratentorial region.

Basic characteristics between patients with and without severe BG-EPVS are given in Table 1. In univariate analysis, vascular risk factors did not differ between two groups. Laboratory tests were comparable between two groups except that patients with severe BG-EPVS had lower level of HbA1c (median: 6.0% vs 6.1%, *p* < 0.05). Diastolic BP (86.20 ± 13.03 mmHg vs 82.61 ± 11.35 mmHg, *p* < 0.05) but not systolic BP at admission was higher in patients with severe BG-EPVS. Considering imaging characteristics, sites of stroke did not differ between two groups. However, patients with severe BG-EPVS had larger infarct size (median: 12.18 mm vs 10.07 mm, *p* < 0.05), more severe WMH (including total WMH, periventricular WMH and deep WMH) and brain atrophy. EPVS in centrum semiovale were comparable between two groups.

**Table 1:** Characteristics between patients with and without severe BG-EPVS.

	Severe BG-EPVS(n = 89)	Controls(n = 178)	*P* value
Demographics			
Age, ^a^ years	76 (67, 79)	76 (67, 79)	0.922
Sex, male	49 (55.1%)	98 (55.1%)	1.000
Vascular risk factors			
Hypertension	70 (78.7%)	121 (68.0%)	0.068
Diabetes	26 (29.2%)	58 (32.6%)	0.576
Hyperlipidemia	18 (20.2%)	28 (15.7%)	0.359
Coronary heart disease	15 (16.9%)	35 (19.7%)	0.579
Stroke or TIA	32 (36.0%)	51 (28.7%)	0.224
Smoking	29 (32.6%)	78 (43.8%)	0.077
Alcohol use	19 (21.3%)	46 (25.8%)	0.420
Systolic blood pressure, mmHg	153.66 ± 20.45	153.29 ± 20.34	0.887
Diastolic blood pressure, mmHg	86.20 ± 13.03	82.61 ± 11.35	0.021 ^b^
NIHSS at admission ^a^	2 (1, 5)	2.5 (1, 4)	0.762
Laboratory tests			
Hemoglobin, g/L	135.66 ± 17.58	133.74 ± 13.86	0.369
Platelets, 10^9^/L	194.91 ± 55.66	207.73 ± 59.74	0.092
Triglyceride, ^a^ mmol/L	1.15 (0.82, 1.64)	1.17 (0.89, 1.68)	0.596
Cholesterol, mmol/L	4.53 ± 1.14	4.64 ± 1.02	0.393
Low density lipoprotein, mmol/L	2.50 ± 0.79	2.58 ± 0.69	0.397
High density lipoprotein, ^a^ mmol/L	1.26 (1.06, 1.57)	1.28 (1.05, 1.51)	0.893
Plasma fibrinogen, mg/dL	301.83 ± 60.70	296.11 ± 68.05	0.503
Hemoglobin A1c, ^a^ %	6.0 (5.7, 6.45)	6.1 (5.8, 7.3)	0.044 ^b^
Homocysteine, ^a^ μmol/L	17 (14.95, 20.50)	16 (14, 20)	0.123
Blood urea nitrogen, ^a^ mmol/L	4.8 (4, 6)	4.8 (4.1, 6.3)	0.398
Serum creatinine, ^a^ μmol/L	81.6 (69.2, 93.45)	83.2 (66.5, 99.1)	0.343
Proteinuria (positive)	7 (7.9%)	11 (6.2%)	0.605
Uric acid, μmol/L	285.86 ± 77.88	282.23 ± 87.86	0.741
Infarct size (DWI), ^a^ mm	12.18 (10.08, 14.98)	10.07 (8.38, 14.16)	0.001 ^b^
Infarct sites			
Subcortical white matter	49 (55.1%)	86(48.3%)	0.518
Basal ganglia	13 (14.6%)	26 (14.6%)	
Thalamus	8 (9%)	27 (15.2%)	
Infratentorial region	19 (21.3%)	39 (21.9%)	
Total WMH ^a^ (Fazekas scale)	5 (4, 6)	4 (2, 5)	0.000 ^b^
Mild (0–2)	9 (10.1%)	29 (18.8%)	
Moderate (3–4)	25 (28.1%)	70 (45.5%)	
Severe (5–6)	55 (61.8%)	55 (35.7%)	
Periventricular WMH ^a^ (Fazekas scale)	3 (2, 3)	2 (1, 3)	0.000 ^b^
Deep WMH ^a^ (Fazekas scale)	2 (2, 3)	1.5 (1, 2)	0.000 ^b^
Brain atrophy ^a^	4 (2, 5)	3 (2, 4)	0.000 ^b^
Mild (0–2)	23 (25.8%)	77 (43.3%)	
Moderate (3–4)	38 (42.7%)	86 (48.3%)	
Severe (5–6)	28 (31.5%)	15 (8.4%)	
EPVS in centrum semiovale ^a^	2 (2, 2.5)	2 (2, 2)	0.365

BG-EPVS indicates enlarged perivascular spaces in basal ganglia; TIA indicates transient ischemic attack; NIHSS, NIH Stroke Scale; DWI, diffusion weighted imaging; WMH, white matter hyperintensities.

^a^ Continuous variables with non-normal distribution are expressed as median (interquartile range).

^b^ *p* < 0.05 between patients with and without severe BG-EPVS.

Logistic regression was performed to determine risk factors for severe BG-EPVS, with vascular risk factors (history of hypertension, diabetes mellitus, coronary artery disease, hyperlipidemia, stroke or TIA, smoking and alcohol use), diastolic blood pressure, level of HbA1c, periventricular WMH, deep WMH, EPVS in centrum semiovale and brain atrophy in the model. We found that brain atrophy (OR = 1.40; 95%CI 1.13, 1.73) and deep WMH (OR = 1.88; 95%CI 1.24, 2.83) were independent risk factors for severe BG-EPVS, while history of hypertension and EPVS in centrum semiovale were not associated with severe BG-EPVS. (Table 2)

**Table 2:** Logistic regression for relative factors associated with severe BG-EPVS.

Variables	B	*p* value	OR (95% CI)
Hypertension	0.515	0.151	1.67 (0.83, 3.38)
Diabetes	-0.066	0.863	0.94 (0.45, 1.97)
Hyperlipidemia	0.781	0.058	2.18 (0.97, 4.91)
Coronary heart disease	-0.136	0.735	0.87 (0.40, 1.92)
Stroke or TIA	-0.253	0.453	0.78 (0.40, 1.50)
Smoking	-0.390	0.288	0.68 (0.33, 1.39)
Alcohol use	-0.091	0.829	0.91 (0.40, 2.09)
Diastolic blood pressure	0.016	0.184	1.02 (0.99, 1.04)
Hemoglobin A1c	-0.207	0.109	0.81 (0.63, 1.05)
Periventricular WMH	0.097	0.669	1.10 (0.71, 1.72)
Deep WMH	0.630	0.003	1.88 (1.24, 2.83)
EPVS in centrum semiovale	0.334	0.098	1.40 (0.94, 2.08)
Brain atrophy	0.337	0.002	1.40 (1.13, 1.73)

TIA indicates transient ischemic attack; WMH indicates white matter hyperintensities; BG-EPVS indicates enlarged perivascular spaces in basal ganglia.

## Discussion

This study mainly focuses on severe BG-EPVS in acute lacunar stroke patients. There are two main findings. First, patients with severe BG-EPVS had lower level of HbA1c and diastolic BP at admission, but presented with larger infarct size, more severe WMH and brain atrophy compared to those without. Second, brain atrophy and deep WMH were independent risk factors for severe BG-EPVS.

The relationship between EPVS and brain atrophy is controversial. In a population-based study, degree of EPVS was not associated with brain atrophy defined by brain parenchymal fraction. [9] In another case-control study, association between severe BG-EPVS and brain atrophy disappeared after correction for the false discovery rate. [7] However, in a study of 298 acute stroke patients, brain atrophy was found to be associated with basal ganglia EPVS after adjusting for vascular risk factors and white matter lesions. [13] In our study, we also found that in lacunar stroke patients brain atrophy was an independent risk factor for severe BG-EPVS. Controversial results may be attributed to different included subjects and heterogeneous assessments on brain atrophy. Unfortunately, there is no consensus on brain atrophy assessment in different studies. A postmortem study has shown that corrected brain weight correlate strongly with presence of EPVS, which is consistent with our results. [17] Therefore, our results add evidence to the hypothesis that ex-vacuo dilatation secondary to shrinkage of cerebral tissue may be associated with development of EPVS in basal ganglia, especially in stroke patients.

Other hypotheses about development of EPVS include increased permeability of arterial wall or blood brain barrier (BBB) and disturbance of the drainage route. In patients with active multiple sclerosis lesions, EPVS correlate with increased permeability of BBB expressed by appearance of contrast-enhancing lesions on MRI. [18] It has been proposed that endothelial inflammation may have contribution to the altered BBB function as marker of inflammation, such as C-reactive protein, is associated with EPVS. [19, 20] In addition, leakage of interstitial fluid and structural changes of microvascular wall may cause obstruction of drainage space and consequent occurrence of EPVS. Increasing evidences have revealed that cerebrovascular amyloid deposition which may be resulted from impaired interstitial fluid drainage is associated with EPVS in centrum semiovale. [21, 22]

EPVS have been considered as a novel marker of SVD because of pathologic finding and their close association with WMH. [23] In a study of 1818 elderly individuals, the degree of EPVS was associated with the volume of WMH and the prevalence of lacunes. [9] In our study, we found that patients with severe BG-EPVS had more severe WMH and deep WMH was an independent risk factor for severe BG-EPVS, which is in agreement with a previous study. [7] We did not investigate the relationship between severe BG-EPVS and lacunes because there is colinearity between the presence of lacunes and history of stroke. In addition, although the rating scale for WMH in our study is widely used with a good sensitivity and reliability, it may be better to define WMH by their volume because of observer bias.

Hypertension has been identified a risk factor for EPVS in basal ganglia. [9, 24] The possible mechanisms have been proposed. Increased intraluminal pressure may cause greater extravasation of fluid through the small arteries into perivascular spaces which is supported by rat experiments in which sustained hypertension could cause increased permeability of endothelial cells and fluid-induced damage to surrounding brain tissue. [25] Moreover, elevated pulsatility in these areas could lead to enlargement of perivascular spaces because of the close proximity of PVS to brain parenchyma. [25] However, relationship between hypertension and severe BG-EPVS were not found in our study, which is in line with an earlier study showing that patients with severe BG-EPVS have similar vascular risk factors to those without. [7] This result should be interpreted with caution as our included subjects were lacunar stroke patients and hypertension is a risk factor for lacunar stroke. In addition, it may be more reasonable to assess the association between ambulatory BP and severe BG-EPVS. In a study of 143 lacunar stroke patients, ambulatory BP level was associated with EPVS in basal ganglia. [26] However, only 27% of patients in this study had > 25 EPVS in basal ganglia. Therefore, future studies are needed to investigate the relationship between severe BG-EPVS and hypertension in general population.

Other risk factors, such as carotid artery stenosis and chronic kidney disease, have been reported to be associated with EPVS. [27, 28] However, we did not find associations between severe BG-EPVS and serum creatinine or blood urea nitrogen. The association between severe BG-EPVS and carotid artery stenosis was not assessed because of our lacunar stroke patients by TOAST classification system. Relationship between severe BG-EPVS and microbleeds was also unclear in this study as microbleeds data were not available. Clinical importance of severe BG-EPVS should be noted. It has been reported in some case reports that severe BG-EPVS are associated with parkinsonism. [8, 29] However, this association was not replicated in a retrospective case-control study. [7] Longitudinal studies evaluating any clinical symptoms caused by severe BG-EPVS are needed.

There are several limitations in our study. First, our study was conducted in a single center. Second, this is a cross-sectional study which prevents us from making causal inference. Third, only lacunar stroke patients were included in our study which may reduce the external validity of our study. Fourth, associations between laboratory tests and severe BG-EPVS should be interpreted with caution because of different admission time. In addition, it may be more reasonable and accurate to assess WMH and brain atrophy by their volume, however, which was limited by our image processing abilities.

## Conclusions

Brain atrophy and deep WMH are independent risk factors for severe BG-EPVS, which supports the hypothesis that brain atrophy may be associated with the development of EPVS in basal ganglia.
